# AssiST: convolutional neural network for analysis of antibiotic susceptibility testing

**DOI:** 10.1093/bioadv/vbag063

**Published:** 2026-02-18

**Authors:** Carmen Li, Sydney Schock, Abigail Costa, Amir Mitchell

**Affiliations:** Department of Systems Biology, University of Massachusetts Chan Medical School, Worcester, MA 01655, United States; Department of Systems Biology, University of Massachusetts Chan Medical School, Worcester, MA 01655, United States; Department of Systems Biology, University of Massachusetts Chan Medical School, Worcester, MA 01655, United States; Department of Systems Biology, University of Massachusetts Chan Medical School, Worcester, MA 01655, United States

## Abstract

**Summary:**

Antibiotic susceptibility testing (AST) is routinely used to evaluate microbial responses to antimicrobials. We present AssiST, a convolutional neural network (CNN) pipeline that classifies microbial growth in scanned 96-well broth microdilution plates to infer drug susceptibility at scale. AssiST accommodates diverse growth morphologies and supports a user-configurable mapping from phenotype to susceptibility calls, enabling flexible use across microorganism species, media types, and drugs. AssiST allows labs to convert flatbed-scanner images into reproducible drug sensitivity readouts with a standard personal computer.

**Availability and implementation:**

AssiST is distributed as a MATLAB library and is freely available for non-commercial use. Code, documentation, and training/inference instructions are available at https://github.com/Mitchell-SysBio/AssiST/. We also provide pre-trained models and a library of sample images. The software accepts image files from standard flatbed scanners. We commit to maintaining the repository for at least 2 years post-publication.

## 1 Background

Antibiotic susceptibility testing (AST) is essential in clinical, research, and educational settings (Kadeřábková *et al.* 2024). For example, in the clinic, antibiotic susceptibility testing is routinely performed on pathogens isolated from patient samples to inform treatment options and guide decisions on which antibiotics should be administered. In the US alone, millions of antibiotic susceptibility tests are performed every year as part of the standard of care during the diagnosis and treatment of bacterial infections such as urinary tract infections and bacteremia. In the setting of basic research, AST is widely used to study drug-response variation across taxa, evolved drug resistance, and nutrient effects on antibiotic efficacy. In educational settings, AST serves as a practical framework for teaching microbial growth principles and data interpretation.

**Figure 1:**
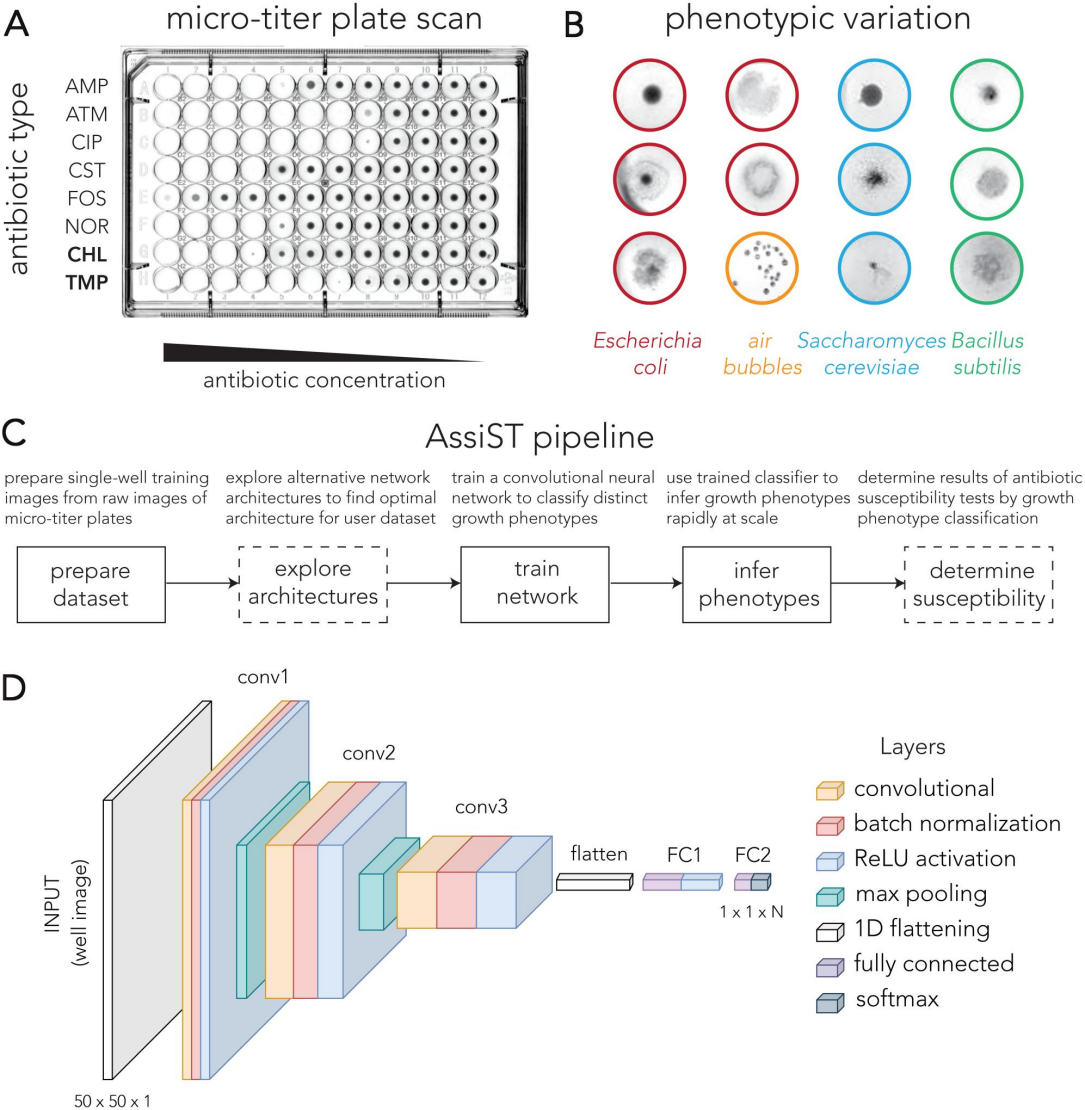
Inference of antibiotic susceptibility with a convolutional neural network. (A) Representative image of antibiotic sensitivity profiles observed in a broth microdilution assay. Two bacteriostatic antibiotics, chloramphenicol (CHL) and trimethoprim (TMP), are marked in bold. The remaining antibiotics, ampicillin (AMP), aztreonam (ATM), ciprofloxacin (CIP), colistin (CST), fosfomycin (FOS), and norfloxacin (NOR), are bactericidal. (B) Representative images of growth phenotypes observed across different microbial species and growth conditions. (C) Overview of the main steps in the AssiST pipeline. Boxes with dashed lines mark optional steps. (D) Diagram of the three-layer convolutional neural network used by AssiST. The specific parameters of each layer, such as filter size and number, can be set by the user.

Broth microdilution assays are commonly used for AST (Fig. 1A). These assays monitor microbial growth in microtiter plates after incubation in the presence of antibiotics dispensed at graded concentrations. Drug susceptibility is inferred after 16–20 h using descriptive endpoints, most commonly the minimum inhibitory concentration (MIC). Depending on the application, these assays may use different organisms, media formulations, incubation temperatures, and growth conditions. Such factors introduce substantial variation in growth dynamics and endpoint phenotypes (Fig. 1B). While automated platforms for reading microdilution assays are available, their cost restricts deployment mainly to centralized clinical laboratories in developed countries that perform testing at scale. In most other settings, results are determined by visual assessment by human experts that considerably limits throughput and can introduce variability between individuals.

Here we present a computational approach for automated analysis of images from broth microdilution tests using a convolutional neural network (CNN). The method can be trained on user-supplied data, enabling evaluation of diverse growth phenotypes and supporting use across microorganisms, media formulations, and growth conditions. Additionally, our approach supports high-throughput classification of bacterial growth in microtiter-plate images acquired with a standard document scanner, providing a cost-effective alternative to dedicated equipment. We demonstrate the value and flexibility of this approach by analyzing susceptibility across multiple antibiotics and conditions.

## 2 AssiST

The AssiST package implements a three-stage MATLAB pipeline for automating AST image analysis: preparation, training, and inference. Figure 1C shows a breakdown of the pipeline. In the preparation step, users curate a training set from raw plate images by extracting individual microwell images and organizing them into folders that match user-defined phenotypic classes. In the training step, a convolutional neural network is fit to classify single-well phenotypes. Figure 1D shows the network architecture AssiST uses. In the inference step, the trained model classifies phenotypes observed in images of new plates. The pipeline includes two additional optional steps. In the explore architectures step, the user can explore a wide range of network architectures to identify the optimal configuration of network parameters for their specific application (e.g. species). Lastly, the user can also use an optional step to determine the antibiotic susceptibility according to the phenotype inference step.

### 2.1 Data structure

The user uploads multiple raw images of broth microdilution plates (similar to the one presented in Fig. 1A). Using the “prepareTrainingSet” script, the images are then cropped into single-well images and are saved to a local folder for manual classification.

Observing the phenotypic variation in single-well images, the user then assigns the cropped images into separate subfolders (named by the distinct classes). To further increase diversity in the dataset, the user can further create four copies of each single-well image by rotating it in 90°, 180°, and 270° angles using the “rotateTrainingImages” script. At the end of this step, the user obtains a training set of class-labeled single-well images. Since performance of machine-learning based classification increases with increased size of the training dataset, large training datasets are recommended. In our experience, a dataset of roughly a thousand single-well images, before rotation, is typically sufficient for most applications.

### 2.2 Explore (optional)

To find the optimal network architecture for the user’s application (e.g. microbial species), the user can test the performance of a large collection of alternative architectures using “explorerCNNparameters.” The script defines this collection by iterating over value ranges for all major network parameters (filter size, learning rate, and number of filters for each layer). The image dataset from the preparation step is randomly partitioned to a training set (80%) and validation set (20%), and this partition is used for training all networks. Since training of alternative networks is independent from one another, the script leverages on parallel computing to speed up this step. Lastly, the script plots the training and validation accuracy of all networks and ranks the alternative networks by their performance.

### 2.3 Train

A single network, with a predefined configuration, is trained on the single-well dataset using the “constructSingleNetwork” script. The image dataset from the preparation step is randomly partitioned to a training set (80%) and validation set (20%). The script plots additional graphs for evaluating the training performance, such as the changes in accuracy, loss as a function of the training iteration (epoch), and the class confusion matrix that allows the user to detect potential pitfalls with the pre-defined classes. Training a single network on a dataset of thousands of images on a standard personal computer is expected to be completed within a few minutes.

### 2.4 Infer

The trained network can be used to infer the phenotypes in new microplate images using the “classifyFullPlates” script. The script infers the phenotype class of every well separately and overlays that phenotype on the plate image. In this step, the user can also provide a plate map showing the antibiotic type and concentration of each well. This map, in turn, is used to derive the susceptibility calls. Inferring phenotypes of a 96-well plate is expected to take a few seconds on a standard personal computer.

## 3 Case study—susceptibility testing across eight antibiotics

We used AssiST to evaluate susceptibility against different antibiotics and compared its performance against two trained human evaluators using the gram-negative *Escherichia coli* F-18 (Cohen *et al.* 1983) and gram-positive *Bacillus subtilis*. For this application we grew the commensal strain of *E. coli* F-18 (Cohen *et al.* 1983) in media supplemented with eight standard antibiotics (two bacteriostatic antibiotics, chloramphenicol and trimethoprim, and six bactericidal antibiotics, ampicillin, aztreonam, ciprofloxacin, colistin, fosfomycin, and norfloxacin). We followed the recommended EUCAST procedure for susceptibility testing by broth microdilution except that defined M9 media was used instead of Mueller Hinton Broth (European Committee for Antimicrobial Susceptibility Testing (EUCAST) of the European Society of Clinical Microbiology and Infectious Diseases (ESCMID) 2003). Briefly, several bacterial colonies inoculated from an agar plate were grown for ∼2 h at 37°C in M9 media and diluted into a 96-well u-shaped bottom plate supplemented with antibiotics (50 µL volume with 5 × 10^5^ cells/ml). The cultures were left to grow for 20 h without shaking in sealed bags in a 37°C incubator. The plate was then removed from the incubator and scanned by a flatbed document scanner (Epson Perfection V750 Pro scanner using photo mode, 8-bit grayscale, and 150 dpi resolution). We recommend users optimize imaging configuration for their own scanner model by inspecting growth phenotypes they detect in the images (e.g. by avoiding over saturation).

We grew *B. subtilis* in Lysogeny Broth (LB) media supplemented with eight standard antibiotics (three bacteriostatic antibiotics, azithromycin, chloramphenicol, and trimethoprim, and five bactericidal antibiotics, ampicillin, ciprofloxacin, fosfomycin, norfloxacin, and vancomycin). We followed a similar procedure to that used for *E. coli* with modifications for optimal growth conditions (incubation at 30°C and incubation time of 24 h).

Before inference, we collected a set of 3,935 single-well images from experiments growing multiple strains of *E. coli* in defined M9 media in the presence of different antibiotics. Observing the phenotypic variation in our images, we chose to assign the single-well images into seven distinct classes (subfolders): pellet and cloud (representing robust growth), small pellet and small cloud (representing restricted growth), tiny pellet (representing marginal growth), empty (representing no detectable growth), and bubbles (representing air bubbles obstruct reading). To further increase diversity in our dataset, we created four copies of each image by rotating them. For *B. subtilis* we collected a set of 1,274 single-well images and assigned the single-well images into four distinct classes (subfolders): cloud (representing robust growth), small cloud and pellet (representing restricted growth), and empty (representing no detectable growth).

**Figure 2:**
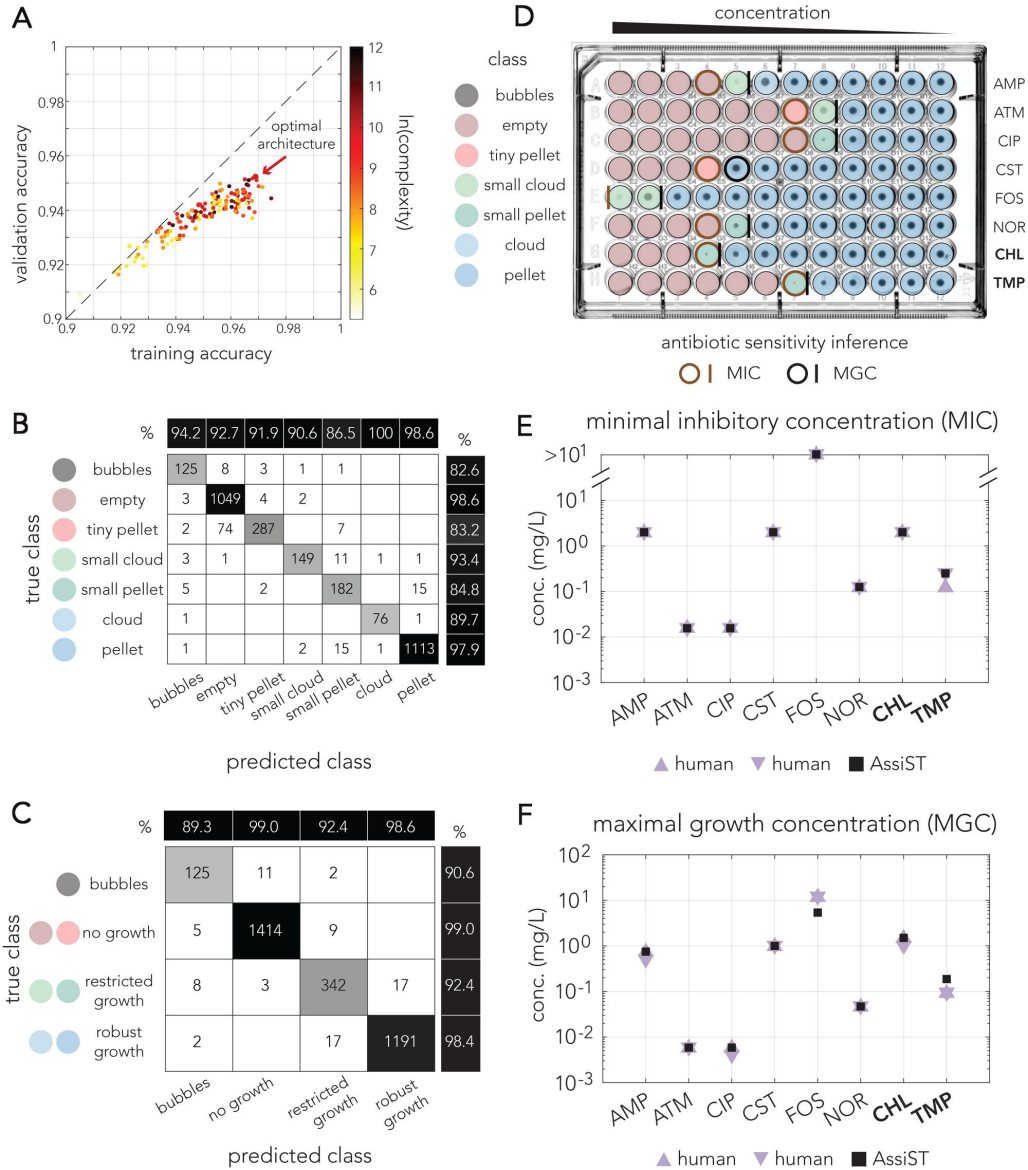
Application of AssiST for antibiotic susceptibility testing in *E. coli*. (A) Network performance of 160 alternative network architectures. The graph shows the network accuracy for the training and validation datasets. The colors mark the network complexity (calculated as the product of the number of filters in each layer and the filter size). (B) Confusion matrix of the optimal network for the validation set. (C) Confusion matrix of the optimal network architecture for grouped classes informative for inferring antibiotic susceptibility. (D) Phenotypes inferred for representative broth microdilution plates. The classes are marked by a color code in panel B. Brown and black circles mark the concentration inferred for minimal inhibitory and maximal growth, respectively. Lines indicate the inferred concentration was between two well concentrations. (E) Minimal growth inhibition values inferred for the broth microdilution plate in panel D by two human evaluators and the AssiST pipeline. (F) Maximal growth concentration values inferred by two human evaluators and the AssiST pipeline.

We explored 160 different alternative network architectures to identify the optimal network architecture for our dataset. All network architectures had three convolutional layers that differed from one another by multiple key parameters (filter size, learning rate, and number of filters for each layer). Figure 2A shows the accuracy of all tested networks across the training and validation dataset. As the figure shows, we observed high performance across all networks, with a median validation accuracy of 0.939. As expected from convolutional neural networks, we also observed positive correlation between accuracy and network complexity (calculated as the product of the number of filters in each layer and the filter size), with more complex networks trending to overfit (training accuracy > validation accuracy). The top performing network had a validation accuracy of 0.948 (configuration: N1 = 4, N2 = 32, N3 = 32, filter size = 5, and learning rate = 0.01). Performance accuracy slightly dropped to 0.933 when we excluded rotated images from the training set. Figure 2B shows the confusion matrix for the optimal classifier. Figure 2C shows a confusion matrix after grouping together classes of similar growth categories.

We used the top classifier to infer the phenotypes of the three replicate plates from our antibiotic susceptibility testing. Figure 2D shows the inferred phenotypes overlayed on the original image of the broth microdilution plate. We then calculated two metrics from these plates, the MIC and the maximal growth concentration (MGC) which represents the highest drug concentration still permitting strong growth. It is important to note that the rules for inferring the MIC for bacteriostatic (growth arresting) and bactericidal (killing) antibiotics are different. Restricted growth is considered sufficient growth for bactericidal drugs, while only robust growth is sufficient for bacteriostatic drugs. We incorporated this rule into our inference. Figure 2E and F shows a comparison between the MIC and MGC determined by two human experts and the inference by AssiST in one of the replicate plates. As the figures show, we observed essential agreement as defined by EUCAST guidelines (within ±1 dilution) between those estimates across all antibiotics.

**Figure 3:**
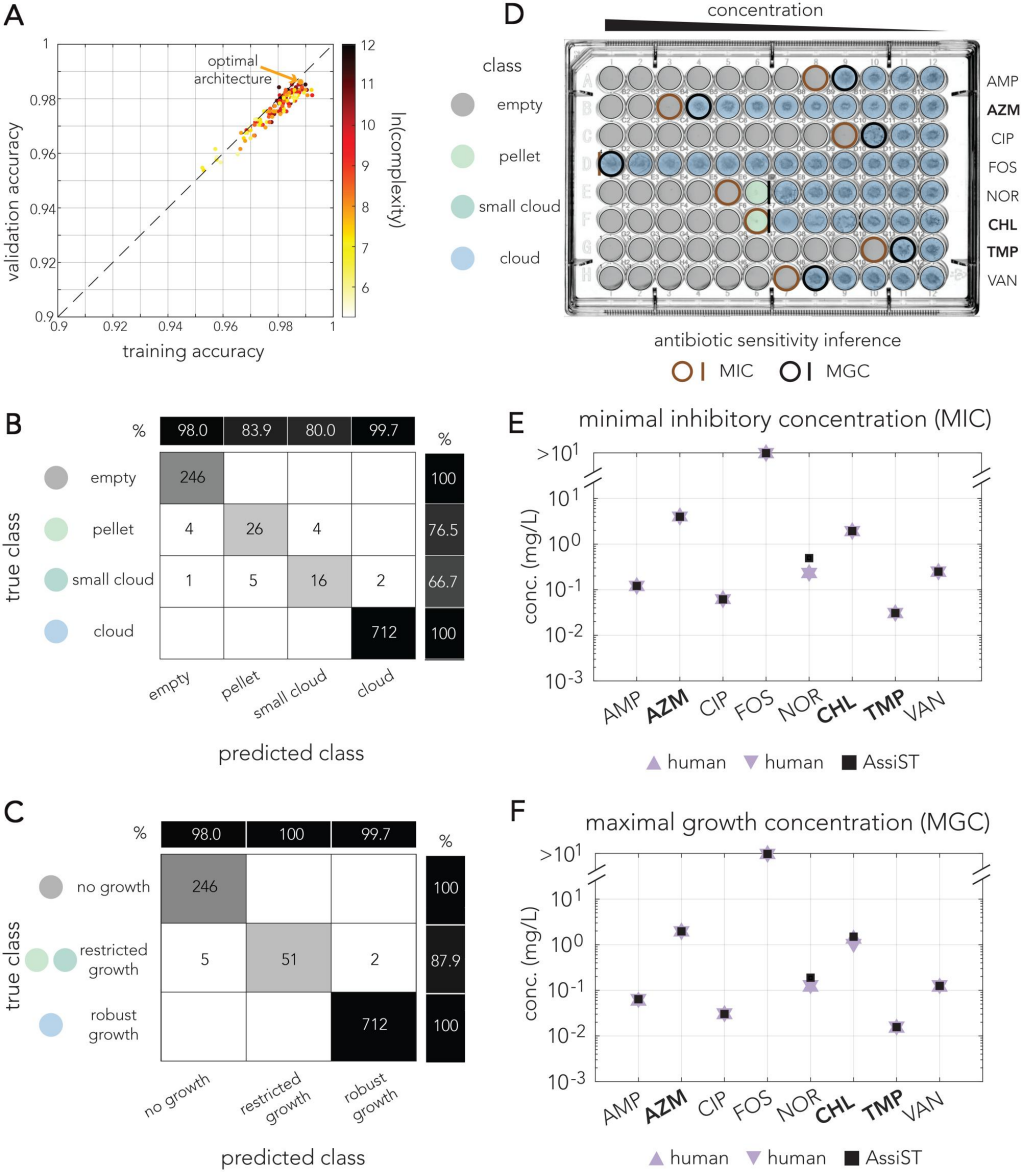
Application of AssiST for antibiotic susceptibility testing in *B. subtilis*. (A) Network performance of 160 alternative network architectures. The graph shows the network accuracy for the training and validation datasets. The colors mark the network complexity (calculated as the product of the number of filters in each layer and the filter size). (B) Confusion matrix of the optimal network for the validation set. (C) Confusion matrix of the optimal network architecture for grouped classes informative for inferring antibiotic susceptibility. (D) Phenotypes inferred for representative broth microdilution plates. The classes are marked by a color code in panel B. Brown and black circles mark the concentration inferred for minimal inhibitory and maximal growth, respectively. Lines indicate the inferred concentration was between two well concentrations. (E) Minimal growth inhibition values inferred for the broth microdilution plate in panel D by two human evaluators and the AssiST pipeline. (F) Maximal growth concentration values inferred by two human evaluators and the AssiST pipeline.

As Fig. 3A shows, for *B. subtilis* we observed high performance across all networks, with a median validation accuracy of 0.979. The top performing network had a validation accuracy of 0.984 (configuration: N1 = 8, N2 = 32, N3 = 32, filter size = 3, and learning rate = 0.01). Performance accuracy slightly dropped to 0.969 when we excluded rotated images from the training set. Figure 3B shows the confusion matrix for the optimal classifier. Figure 3C shows a confusion matrix after grouping together classes of similar growth categories. Figure 3E and F shows a comparison between the MIC and MGC determined by two human experts and the inference by AssiST in one of the replicate plates. As the figures show, we observed essential agreement (within ±1 dilution) between those estimates across all antibiotics.

## 4 Conclusion

AssiST is an open-source MATLAB pipeline that uses a convolutional neural network to classify bacterial growth phenotypes from microtiter-plate images. Running on modest, widely available resources, a standard personal computer and a flatbed document scanner, it enables rapid, scalable antibiotic susceptibility testing. It should be noted that our method imposes discrete categories on growth phenotypes sampled from a continuous morphological space. These categories, in turn, are subjectively defined by the human assembling the training set. We therefore expect that some of the classification errors arise from borderline phenotypes that are positioned between neighboring categories in the morphological space. Since classification of such ambiguous phenotypes is subjective, we expect similar disagreement will also exist among human classifiers. Indeed, we observed that most errors occurred from misclassification of growth phenotype to a neighboring morphological class, e.g. “tiny pellet” wells mis-classified as “empty” wells (Fig. 2B). To help users easily identify potentially erroneous classifications we included in the code an option to flag wells with classification probabilities below a threshold of posterior probability.

Our implementation offers three practical advantages: (i) user-defined phenotypic classes beyond binary growth/no-growth, allowing a single trained model to accommodate diverse phenotypes that are expected to arise when working across different bacterial species and antibiotic classes. This flexibility is notable given previous work that implemented a similar deep learning-based framework exclusively for testing *Mycobacterium tuberculosis* using a single format image type (Vo *et al.* 2025), (ii) Support for assays beyond AST, including studies of drug influence at subinhibitory concentrations on cellular phenotypes (Kals *et al.* 2025) that may manifest in bulk culture phenotypes and even temporal growth arrest phenotypes (Li *et al.* 2025), and (iii) an optional module for comparing alternative network architectures and reporting performance, making it straightforward to substitute a model better suited to a specific dataset. Together, these features lower the barrier to adoption and adaptation across clinical, research, and educational settings.

## Data Availability

The data underlying this article are available in *Github* at https://github.com/Mitchell-SysBio/AssiST/.
